# Single-cell differences in matrix gene expression do not predict matrix deposition

**DOI:** 10.1038/ncomms10865

**Published:** 2016-03-03

**Authors:** Allison J. Cote, Claire M. McLeod, Megan J. Farrell, Patrick D. McClanahan, Margaret C. Dunagin, Arjun Raj, Robert L. Mauck

**Affiliations:** 1 Department of Bioengineering, University of Pennsylvania, Philadelphia, Pennsylvania 19104, USA; 2 Department of Orthopaedic Surgery, McKay Orthopaedic Research Laboratory, Perelman School of Medicine, University of Pennsylvania, Philadelphia, Pennsylvania 19104, USA; 3 Translational Musculoskeletal Research Center, Philadelphia VA Medical Center, Philadelphia, Pennsylvania 19104, USA

## Abstract

Mesenchymal stem cells (MSCs) display substantial cell-to-cell heterogeneity, complicating their use in regenerative medicine. However, conventional bulk assays mask this variability. Here we show that both chondrocytes and chondrogenically induced MSCs exhibit substantial mRNA expression heterogeneity. Single-molecule RNA FISH to measure mRNA expression of differentiation markers in single cells reveals that sister cell pairs have high levels of mRNA variability, suggesting that marker expression is not heritable. Surprisingly, this variability does not correlate with cell-to-cell differences in cartilage-like matrix production. Transcriptome-wide analysis suggests that no combination of markers can predict functional potential. De-differentiating chondrocytes also show a disconnect between mRNA expression of the cartilage marker aggrecan and cartilage-like matrix accumulation. Altogether, these quantitative analyses suggest that sorting subpopulations based on these markers would only marginally enrich the progenitor population for ‘superior' MSCs. Our results suggest that instantaneous mRNA abundance of canonical markers is tenuously linked to the chondrogenic phenotype at the single-cell level.

## Abstract (web summary)

Regenerative tissue engineering with mesenchymal stem cells is hampered by bulk methods of assessing differentiation status and a general assumption that expression of individual markers of stem cell differentiation correlate with functional capacity. Here the authors debunk this assumption by applying single-cell techniques to disassociate aggrecan mRNA abundance and matrix deposition.

## Graphical abstract

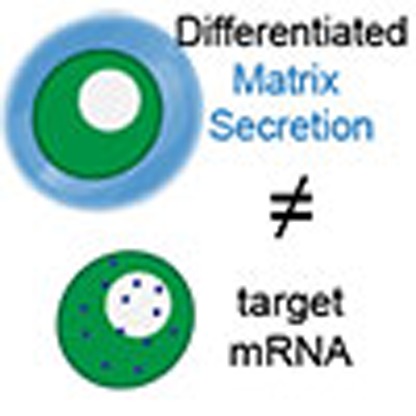

Regenerative medicine strategies such as tissue engineering combine advances in cell biology, biomaterials and medicine to restore tissue function. Some approaches utilize stem cells for regeneration. For example, researchers commonly use multipotent progenitor cells, including mesenchymal stem cells (MSCs), for tissue engineering due to their capacity to undergo either osteogenic, adipogenic or chondrogenic differentiation^1^. However, even with the most effective differentiation protocols, individual MSCs demonstrate heterogeneity in their biophysical properties and in their ability to undergo lineage commitment^2^,^3^,^4^,^5^, with some clonal subpopulations robustly committing to a differentiated fate while other clones fail to respond to differentiation cues^3^,^6^,^7^. Furthermore, in cases in which it seems as though all cells have differentiated based on bulk expression of a particular marker, individual cells within the population may continue to express markers of other lineages^8^,^9^. Given that underperforming, alternatively performing or non-responsive subpopulations will hinder the performance of engineered tissues, this inherent MSC heterogeneity compromises therapeutic efficacy. As such, quantitative strategies to select ‘superior' subpopulations *a priori* would improve translational potential.

Despite the phenotypic heterogeneity in MSC populations, most studies that explore the molecular underpinnings of phenotype monitor differentiation via bulk assays of transcriptional state and protein synthesis averaged over an entire cell population. These ensemble measurements, by definition, mask population heterogeneity^10^,^11^. The advent of single-cell methods allows for the measurement of cell-to-cell variation and the ability to quantify absolute gene expression in a single cell^12^,^13^,^14^, revealing, for example, marked transcriptional heterogeneity. Real-time fluorescent monitoring of changes in transcript levels in individual cells has also shown that individual MSCs differ in the timing and extent to which they upregulate an early osteogenic marker^15^. These findings underscore the limitations of coarse ensemble approaches and highlight the need for single-cell molecular profiling of these differentiation events. Although it is reasonable to speculate that the subpopulation of cells expressing high levels of marker genes would ultimately be the most chondrogenic, this hypothesis remains untested.

Given that individual MSCs are highly variable in their capacity to undergo chondrogenesis and accumulate cartilage-like matrix^16^, we postulated that one could use single-cell marker gene transcript levels as a means to enrich for MSC subpopulations most suited for therapeutic application. Here we define this relationship by developing probe sets for RNA fluorescence *in situ* hybridization (FISH) directed against transcripts of markers of cartilage, bone and fat, and use single-cell analysis to delineate the relationships between absolute transcript level and differentiated cell function. Specifically, we hypothesized that cells that robustly accumulate an aggrecan-rich, cartilage-like matrix would also express high levels of aggrecan mRNA, while at the same time suppressing markers of other lineages.

We find surprising levels of variability in the expression of aggrecan and other marker genes between individual MSCs both before and after differentiation. However, when we compare the expression with functional capacity (defined by actual matrix deposition) on a single-cell basis, we find a weak correlation between transcript abundance and protein expression. Transcriptome-wide analysis via RNA sequencing further suggests that neither an expanded set of marker genes, nor the principal components of global gene expression variation, correlate strongly with functional capacity. Indeed, even in fully differentiated chondrocytes derived from native tissue, absolute aggrecan mRNA expression is decoupled from cartilage-like matrix accumulation. Collectively, these findings suggest that sorting based solely on a small set of differentiation markers will not improve chondrogenic outcomes, and challenge the traditional notion that marker gene expression defines or is even strongly associated with phenotype.

## Results

### Single cells express differentiation markers heterogeneously

**Figure 1:**
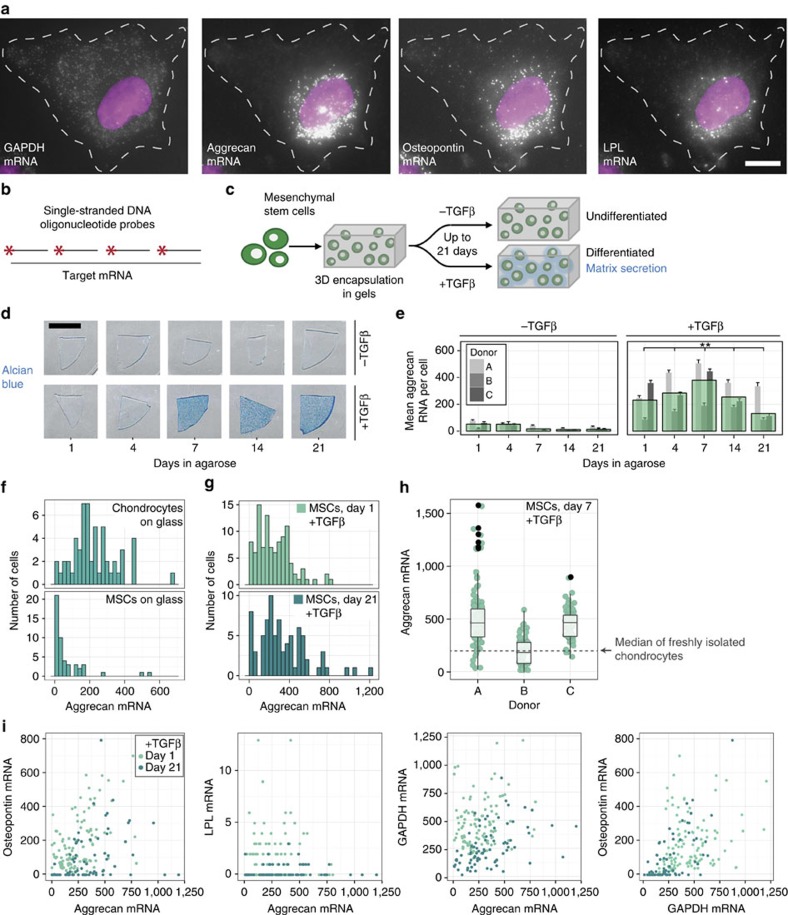
RNA FISH reveals heterogeneity in lineage marker expression in MSCs and chondrocytes. (**a**,**b**) Representative images (**a**) and schematic (**b**) of single-molecule RNA FISH, in which fluorescently labelled DNA oligonucleotides enable quantification of absolute expression of multiple genes in the same cell. Scale bar, 10 μm. (**c**) Chondrogenic induction scheme, involving cell encapsulation in 3D agarose constructs and exposure to TGFβ. (**d**) Alcian blue staining for sulfated proteoglycans; Donor B shown. Scale bar, 5 mm. (**e**) Mean aggrecan RNA counts in MSCs cultured in 3D for up to 21 days. Narrow bars represent the mean within an individual donor; overlaid bars represent the mean across donors. Error bars indicate standard error (*n*=24–128 cells per donor and condition). Means compared by *t*-tests with Satterthwaite approximation and simulated adjustment for multiple comparisons, **P<0.01 versus −TGFβ conditions, and between +TGFβ time points. See Supplementary Table 4 for all statistical comparisons. (**f**,**g**) Distributions of single-cell aggrecan expression for chondrocytes and MSCs plated on glass in basal media (**f**, *n*=56 chondrocytes, 49 MSCs) and 3D encapsulated MSCs exposed to TGFβ for 1 and 21 days (**g**, *n*=105 cells for day 1, 79 cells for day 21; Donor A shown.) (**h**) Single-cell aggrecan expression for each donor after 7 days of 3D culture with TGFβ relative to the median aggrecan expression in freshly isolated chondrocytes (dashed line; *n*=103 cells for Donor A, 54 cells for Donor B and 65 cells for Donor C). (**i**) Simultaneous expression of aggrecan, osteopontin, LPL and GAPDH on day 1 and day 21; Donor A shown (*n*=105 cells for day 1, 79 cells for day 21).

To quantify absolute gene expression of marker genes on a single-cell basis during MSC differentiation and chondrocyte de-/re-differentiation, we paired classic cartilage tissue engineering and cartilage biology experiments with single-molecule RNA FISH^17^,^18^. Specifically, we monitored the simultaneous expression of aggrecan as a marker of chondrogenic differentiation, GAPDH as a reference gene, and osteopontin and lipoprotein lipase (LPL) as markers of alternate fates (osteogenesis and adipogenesis, respectively)^19^,^20^,^21^. For each gene, we designed fluorescently labelled sequence-specific oligonucleotide probes to visualize individual mRNA molecules in intact fixed cells. Individual mRNA appeared as bright diffraction limited spots (Fig. 1a,b), and subsequent spot counting yielded absolute copy number at the single-cell level.

To show that our measurements corresponded well with existing measurements of these systems, we first determined how absolute gene expression changed as MSCs underwent chondrogenic differentiation. To do so, we formed engineered constructs and used RNA FISH to quantify gene expression over 3 weeks in chemically defined media with or without transforming growth factor-β (TGFβ; chondrogenic induction media and control media, respectively, Fig. 1c). As expected, chondrogenic induction promoted proteoglycan synthesis and matrix accumulation (Fig. 1d) and increased aggrecan copy number in comparison with control media (Fig. 1e). Although there was considerable donor-to-donor variability in mean aggrecan levels and matrix deposition, the trends were similar between donors, with mean aggrecan copy number generally increasing over the first 7 days, before decreasing at later time points (Fig. 1e, Supplementary Fig. 1A and Table 1). Mean GAPDH copy number increased with exposure to induction media (Supplementary Fig. 1b). Thus, in aggregate, this RNA FISH analysis aligned with the canonical understanding of gene expression changes during chondrogenic differentiation^22^.

While these ensemble measures corresponded with previous findings, they did not provide information on cell-to-cell variability in expression of these lineage markers. Thus, we measured mRNA copy number on a cell-by-cell basis under baseline conditions and with differentiation. We assayed four conditions: naive MSCs in expansion culture, MSCs differentiating in engineered constructs after 1 and 21 days in induction media, and as a positive control, fully differentiated primary chondrocytes (Fig. 1f,g). For each of these groups, single-cell analysis showed striking heterogeneity in expression, with aggrecan mRNA copy number per cell spanning three orders of magnitude (10^0^–10^2^). Consistent with the notion that stem cells exhibit greater variability than differentiated cells, naive MSCs showed the greatest heterogeneity in aggrecan expression (as measured by the coefficient of variation, Table 1), and the coefficient of variation decreased with exposure to induction media. However, the variability remained high even after long periods of time in differentiation culture (Fig. 1g). Fully differentiated chondrocytes had the most homogeneous aggrecan expression of all the cell types and conditions we examined, though their mean aggrecan copy number was slightly lower than that of differentiated MSCs. These data show that MSCs exhibit substantial cell-to-cell expression heterogeneity and that, while chondrogenic culture promotes a chondrocyte-like gene expression pattern, copy number remains highly variable between cells. Indeed, this variability within a population of differentiated MSCs overshadowed differences in mean expression between donors (3–4 orders of magnitude versus a maximum of ∼twofold difference, Fig. 1h).

This heterogeneity may either reflect different subpopulations that have adopted distinct fates or appear in cells that remain uncommitted. In the former scenario, if differentiated MSCs can express markers for only one fate at a time, then alternate lineage commitment should manifest as an anti-correlation between aggrecan and other lineage markers at the single-cell level. To determine whether this was the case, we performed RNA FISH for aggrecan, osteopontin and LPL in the same cells, with the latter two markers indicating osteogenic and adipogenic lineages, respectively. Rather than identifying subpopulations that were distinctly chondrogenic or osteogenic, we instead observed a slight positive correlation between aggrecan and osteopontin (Fig. 1i, Day 1 *ρ*=0.49, *P*<0.001; Day 21 *ρ*=0.34, *P*<0.005). Conversely, LPL expression was minimal, and did not correlate with either aggrecan or osteopontin expression (Supplementary Table 2). These data suggested that heterogeneity in marker expression after differentiation is not due to alternate lineage commitment, but rather highlights the fact that even differentiated MSCs can express high levels of markers for inappropriate lineages.

### RNA levels poorly predict single-cell functional potential

**Figure 2:**
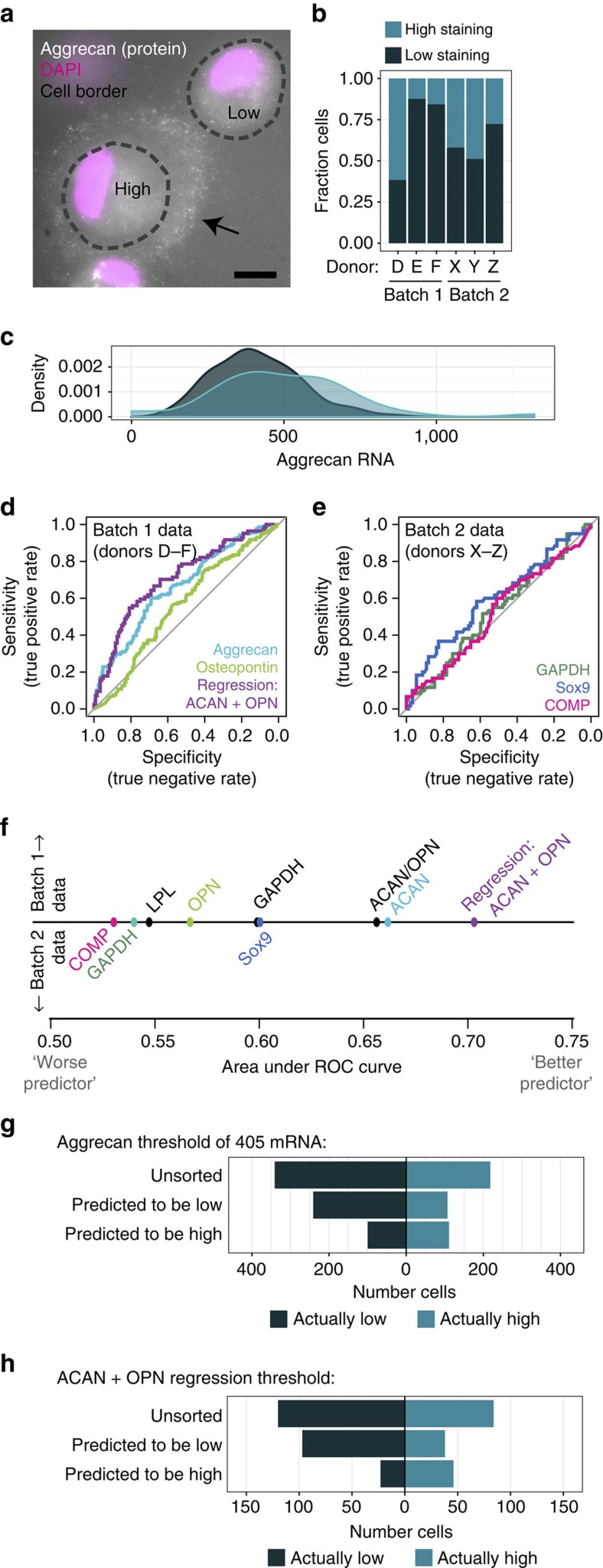
Marker gene expression is a poor predictor of cartilage-like matrix production in individual MSCs. (**a**) Aggrecan core protein identified by immunostaining of MSCs showing high or low cartilage-like matrix formation after 7 days of 3D culture with TGFβ. Scale bar=10 μm. (**b**) Fraction of cells classified as high- or low-performing based on aggrecan protein staining (cells/donor: D: 78, E: 89, F: 51, X: 62, Y: 43, Z: 47). (**c**) Distribution of aggrecan copy number in high- and low-performing MSC populations; probability density curve for Donor E shown (*n*=153 cells). (**d**,**e**) Receiver operating characteristic curves using individual gene expression and regression analysis on combinations of genes from batch 1 (**d**) and individual gene expression from batch 2 genes (**e**) to distinguish between high- and low-performing MSCs (cells/donor: D: 132, E: 153, F: 122, X: 57, Y: 42, Z: 47). (**f**) Summary graph of area under the curve of receiver operating characteristic curves for individual gene expression, gene expression ratios, and regression analysis of combinations of gene. (**g**,**h**) Simulated sorting of MSCs into anticipated high- and low-performing cells, using the optimized threshold of 405 aggrecan mRNA copies (**g**) and the optimized threshold from the ACAN+OPN regression (**h**).

On the basis of this tremendous cell-to-cell heterogeneity in chondrogenic gene expression, we next asked whether aggrecan or other markers might serve as a means for separating robustly chondrogenic cells from the less chondrogenic ones in the initial heterogeneous population. For this to be possible, mRNA levels would need to correlate with chondrogenic capacity, indicated by the accumulation of a proteoglycan-rich extracellular matrix. To determine whether such a connection existed, we seeded MSCs in three-dimensional (3D) culture and induced chondrogenesis for 7 days, the point at which mean aggrecan expression peaked. We then performed immunofluorescent staining for aggrecan core protein (a central component of the cartilage-like extracellular matrix) simultaneously with RNA FISH using one of two probe sets: probes for markers of multiple fates (aggrecan, osteopontin, LPL and GAPDH; Batch 1 samples) or probes for chondrogenic markers (Sox9, cartilage oligomeric matrix protein (COMP) and GAPDH; Batch 2 samples). We designated cells with evidence of extracellular staining for aggrecan core protein as ‘high-performing' (comprising 12–62% of the population, depending on donor), and cells lacking extracellular staining as ‘low-performing' (Fig. 2a,b). Surprisingly, aggrecan expression did not strongly predict aggrecan core protein accumulation. Indeed, even within a single donor, the distribution of aggrecan mRNA abundance in high- and low-performing cells overlapped substantially (Fig. 2c). The mRNA/cell distributions of other chondrogenic markers (COMP, Sox9), markers of alternative fates (osteopontin, LPL) and the housekeeping gene *GAPDH* (Supplementary Fig. 2a) also demonstrated similar overlap. While in aggregate, the high-performing cells had a greater mean expression of aggrecan, COMP and Sox9, and lower mean expression of osteopontin than low-performing cells, the magnitude of these differences was small and similar to the shift seen in GAPDH expression (Supplementary Fig. 2a, aggrecan: 1.35-fold increase, COMP: 1.14-fold increase, Sox9: 1.33-fold increase, GAPDH: 1.17-fold increase, osteopontin: −1.22-fold decrease). We also determined the expression ratios relative to commonly used normalization genes (that is, aggrecan/GAPDH) or to genes indicating alternate lineage specification (that is, aggrecan/osteopontin). These metrics also showed substantial overlap and small effect size (Supplementary Fig. 2a). Thus on this qualitative basis, neither absolute nor normalized single-cell expression of marker genes was highly predictive of chondrogenic capacity at the single-cell level.

To quantify the ability of transcript abundance to predict the extent of a cell's matrix accumulation, and thus sort high- from low-performing cells, we constructed receiver operating characteristic (ROC) curves to determine the ‘true positive' (sensitivity) and ‘true negative' (specificity) rates associated with potential mRNA thresholds. We pooled data across donors assayed using the same probes (batches 1 and 2). Within each batch, we assessed the high/low classification performance of individual genes, gene expression ratios and linear combinations of gene expression levels (Fig. 2d–f, Supplementary Fig. 2b). While each metric discriminated between high- and low-performing cells better than random chance (represented by the diagonal line on the ROC plots, and an area under the curve=0.5), the improvements in selection specificity were relatively small. Of the individual RNA types indicative of the chondrogenic lineage, aggrecan and Sox9 were best able to discriminate between high- and low-performing cells. For example, consider the optimized threshold of 405 aggrecan mRNA, which maximizes the Youden J statistic (sensitivity+specificity−1). Conceptually, we can designate all cells with >405 aggrecan RNA as anticipated high performers, and others as anticipated low performers. For the donors studied, this unsorted population was 34% high- and 66% low-performing cells. Sorting based on this optimized aggrecan threshold misclassified 37% of all cells (that is, percent of high cells predicted to be low, or low cells predicted to be high). 50% of high-performing cells were lost due to incorrect classification as ‘anticipated-low' cells, and the fraction of high-performing cells in the ‘anticipated-high' population was enriched only 35% over the unsorted population (Fig. 2g). A logistic regression model combining aggrecan and osteopontin expression improved on this performance only slightly, where its optimized threshold yielded a 33% misclassification rate, and enriched the fraction of high cells by 37% (Fig. 2h, versus 35% for aggrecan alone). Of the gene expression ratios, aggrecan/osteopontin was a better discriminator than aggrecan/GAPDH, though its selection performance did not surpass that of aggrecan alone. Sorting on a donor-by-donor basis was similarly ineffective (Supplementary Fig. 2b). Thus, sorting cells based on expression of aggrecan, other common differentiation markers, and linear combinations thereof would result in only marginal enrichment of the population, while substantially reducing available cell number.

### Transcriptomics does not identify better marker sets

**Figure 3:**
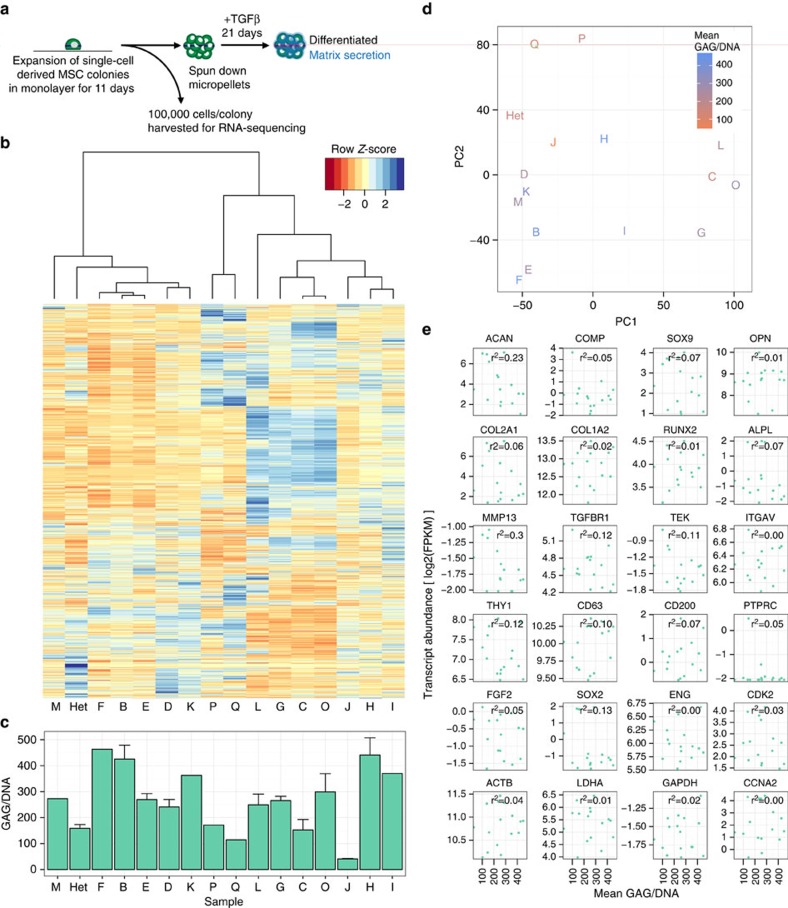
Genome-wide transcriptome profiling does not predict MSC functional potential. (**a**) Schematic for RNA sequencing and testing of functional capacity of single-cell-derived clones. (**b**) Unbiased clustering of clones (and heterogeneous population) based on fragments per kilobase of transcript per million reads (FPKM) of RNA sequencing results (subsetted for genes where at least one sample had FPKM >1). (**c**) Glycosaminoglycan deposition per DNA in micropellets derived from clonal or heterogeneous populations (from part **b**) cultured for 21 days in chondrogenic (TGFβ+) culture media. For clones with limited cell number, one pellet was formed and assayed (Clones M, F, K, P, Q, W, I). For clones with cell number sufficient for multiple pellets, error bars indicate standard deviation (3 pellets—Het and clones B, E, L, G, C, O, J, H; 2 pellets—clone D). (**d**) Principal component analysis of same RNA sequencing results as in part b, coloured by GAG/DNA for each clone. (**e**) Log2 transformed FPKM of selected genes from RNA sequencing results as a function of GAG/DNA for each clone.

On the basis of the inability of aggrecan and other lineage-specific markers to robustly predict matrix accumulation at the single-cell level, we next utilized high-throughput RNA sequencing to determine whether other features of the transcriptome, and specifically factors present in the undifferentiated population, might prospectively identify MSCs with high differentiation potential. We expanded single-cell-derived MSC colonies in monolayer, and collected a fraction of the cells for RNA sequencing and subsequent transcriptome analysis. The remaining fraction was expanded through an additional passage, formed into pellets and cultured in the presence of TGFβ for 21 days to assay chondrogenic potential (Fig. 3a). This evaluation of baseline MSC gene expression in clonal populations derived from single cells had the potential to identify markers that could be used to sort freshly isolated MSCs based on their gene expression signatures.

An initial comparison of differential expression between clones (Fig. 3b), as compared with the deposition of extracellular matrix components of each clone (Fig. 3c), revealed no striking patterns of gene expression that correlated with subsequent matrix deposition. We also used principal component analysis to determine whether the variation between the gene expression of each clone could be used to predict functional capacity, but there was no relationship between clustering in either of the first two principal components and matrix deposition (Fig. 3d).

Given that the full transcriptome lacked global predictive capacity, we next sought to broaden our conclusions from the FISH experiments by examining the sequencing data associated with individual genes. We selected a small subset of genes that corresponded to four categories of markers identified in previous studies: chondrogenic markers, stemness markers, cell cycle-associated genes and housekeeping genes^23^. Consistent with our single-cell analysis results, none of these genes correlated strongly with functional potential on a clonal basis (Fig. 3e). Even the most predictive genes, MMP13 and aggrecan, correlated only weakly (*r*^2^=0.3, *P*=<0.05 and *r*^2^=0.23, *P*=0.062, respectively). Altogether, this transcriptomic analysis suggests that there is no expression signature at the RNA level that could pre-identify specific clones with high chondrogenic potential.

### Marker heterogeneity emerges rapidly after cell division

**Figure 4:**
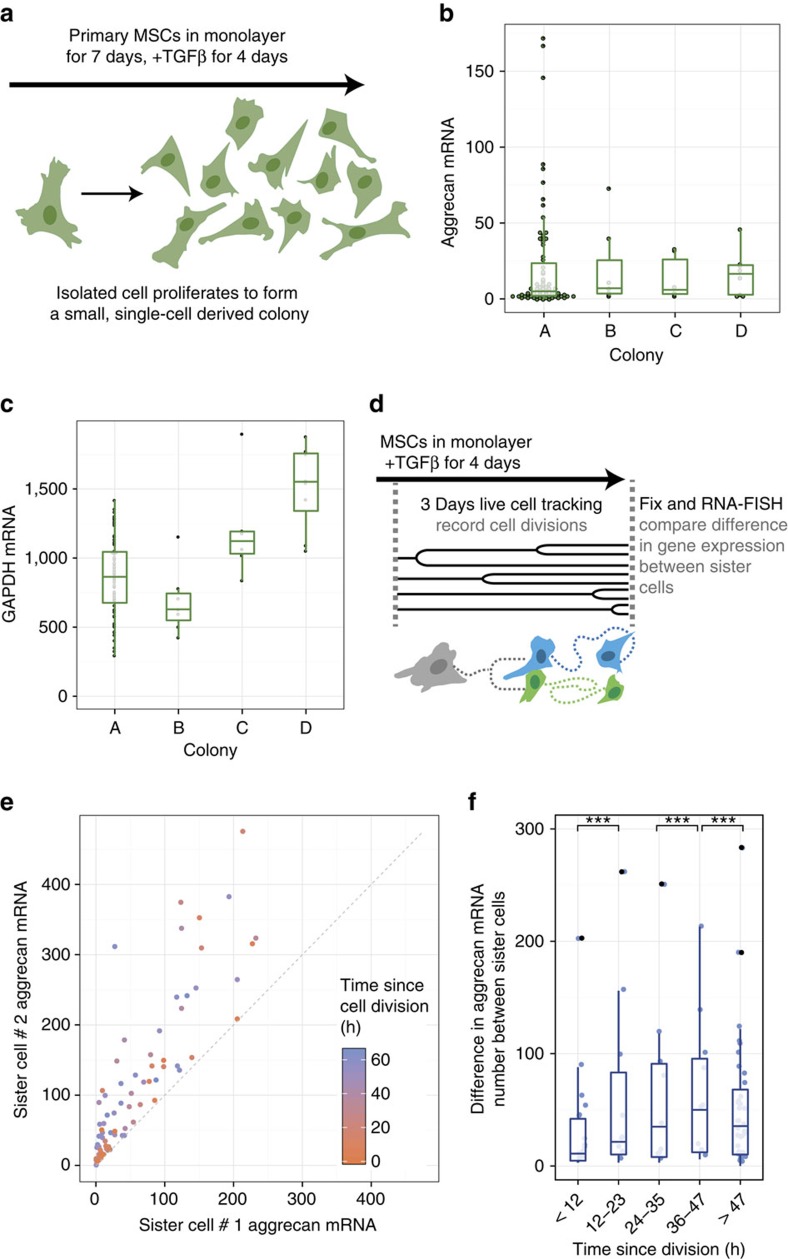
Marker expression heterogeneity emerges shortly after cell division. (**a**–**c**) Gene expression in small MSC colonies. (**a**) Colony formation scheme. (**b**,**c**) Aggrecan and GAPDH expression in four colonies established from a single donor (*n*=75 cells in colony A, 7 cells in colony B, 6 cells in colony C, 8 cells in colony D). (**d**) Live cell tracking scheme to identify sister cell pairs at various times post-cell division. (**e**,**f**) Divergence in aggrecan gene expression between sister cells as a function of time since last division (*n*=81 sister cell pairs). Box hinges denote the first and third quartiles. Whiskers extend from the hinges to the most extreme data points within (1.5 * interquartile range) of the hinges. Means compared by *t*-tests with Satterthwaite approximation and simulated adjustment for multiple comparisons, ***P<0.001, see Supplementary Table 5 for all comparisons.

On the basis of the inability of transcript levels to robustly predict matrix forming potential, we next asked whether it was propagated through cell division; that is, whether cells with a higher expression level would transmit this feature to their daughter cells. As an initial assay, we measured aggrecan copy number in every cell located within a series of small MSC colonies stimulated with TGFβ (where each individual colony likely arose from a single cell, Fig. 4a). Results from this analysis showed that aggrecan copy number varied more within a single colony than it did between colonies (Fig. 4b). This result suggests that with just a few cell divisions, aggrecan levels rapidly devolved to recapitulate the heterogeneity present in the bulk population. In contrast, GAPDH was less variable than aggrecan within each colony (lower coefficient of variation, Supplementary Table 3), but showed greater differences in mean level between colonies (Fig. 4c). Thus, not every gene demonstrated the high intra-colony variability observed in aggrecan expression, and some genes were differentially expressed between colonies. However, without live cell time-lapse measurements of the cellular lineage, it was difficult to directly show that variability in aggrecan mRNA levels arose through randomization rather than heritable differences.

To overcome this limitation, we next continuously tracked MSCs as they migrated and divided in induction media by live cell microscopy for 3 days, and correlated terminal aggrecan expression between sister cells with respect to the time since their last division (Fig. 4d). Shortly after division (<12 h), sister cells had comparable aggrecan and GAPDH levels (Fig. 4e,f, Supplementary Fig. 3a,b), suggesting symmetric partitioning of RNA. However, after more than ∼12 h since division, sister cells showed increasingly divergent levels of aggrecan and GAPDH expression (Fig. 4e,f, Supplementary Fig. 3a,b, Supplementary Table 6). Within cell pairs, aggrecan and GAPDH divergence only weakly correlated, suggesting that the relative difference between sister cells was not globally regulated, underscoring the fact that aggrecan and GAPDH do not necessarily change together (Supplementary Fig. 3c,d). These findings may reflect a difference in cell function as a consequence of asymmetric cell division (that is, sister cells have different target expression levels) or could simply identify how asynchronous dynamic fluctuations lead to temporal differences in expression level. In either case, these differences suggest that a sorted population of high-aggrecan cells would not remain so for more than a couple of days, and may explain why, at the single-cell level, cells with high-aggrecan RNA expression are not necessarily the cells with the greatest amount of matrix deposition.

### Marker genes do not identify a chondrocyte phenotype

**Figure 5:**
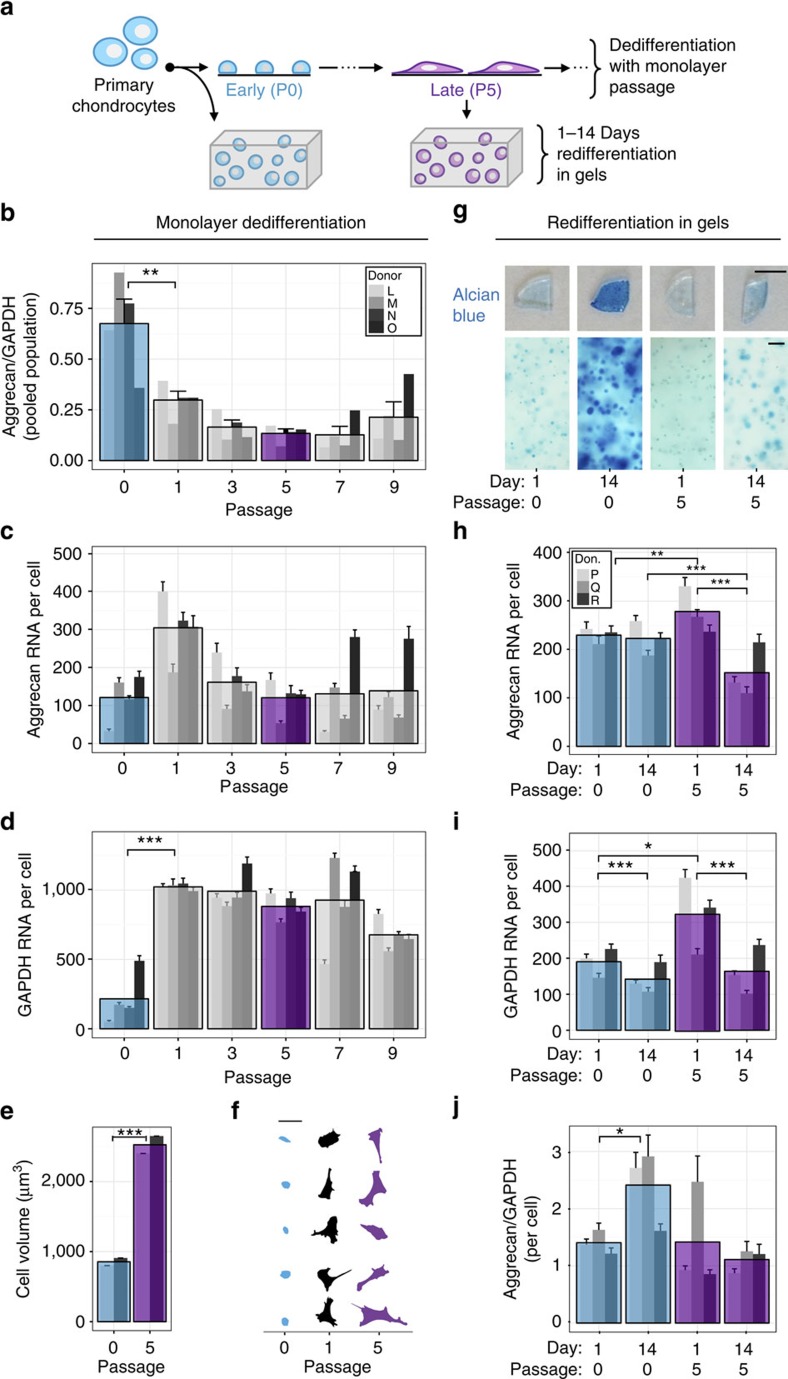
Chondrocyte de- and re-differentiation are not driven by altered absolute aggrecan expression. (**a**) Chondrocyte de-differentiation and re-differentiation scheme. (**b**–**f**) Analysis of chondrocytes de-differentiating with passage in monolayer culture. (**b**) RNA FISH counts of aggrecan pooled over the population and normalized to GAPDH expression. (**c**) Absolute aggrecan expression with passage number. (**d**) Absolute GAPDH expression with passage number (*n*=39–113 cells per donor per passage). (**e**,**f**) Chondrocyte suspended cell volume (**e**) and morphology (**f**) with passage (*n*=274–543 cells per donor per passage). Scale bar, 50 μm. (**g**–**j**) Analyses of early passage (P0) and late passage (P5) chondrocytes re-differentiating in 3D culture. (**g**) Alcian blue staining after 1 and 14 days of 3D culture. Top scale bar, 5 mm, bottom scale bar, 100 μm. (**h**) Absolute aggrecan expression. (**i**) Absolute GAPDH expression. (**j**) Single-cell aggrecan expression normalized to GAPDH expression. (*n*=46–65 cells per donor per condition). Narrow bars represent the mean within an individual donor; overlaid bars represent the mean across donors. Error bars indicate standard error. RNA count means compared by *t*-tests with Satterthwaite approximation and simulated adjustment for multiple comparisons. Pooled aggrecan/GAPDH expression data, cell area data, and cell volume data compared using a one-way analysis of variance with Tukey's post-hoc test. *P<0.05, **P<0.01 and ***P<0.001, see Supplementary Tables 7 and 8 for all comparisons.

While aggrecan gene expression did not correlate with matrix deposition in MSCs, it is a canonical feature of the differentiated chondrocyte ‘phenotype' and is widely considered to be a leading indicator of cartilage-specific extracellular matrix deposition (for example, aggrecan core protein)^24^,^25^,^26^. It is also well accepted that, on serial passaging and expansion in monolayer, chondrocyte matrix production decreases along with a multi-fold decrease in the aggrecan/GAPDH ratio (Fig. 5a)^27^,^28^,^29^,^30^,^31^. This change in expression is associated with increases in cell size and proliferation rate^32^,^33^,^34^. To reconcile our finding of discordant aggrecan expression and matrix deposition in MSCs with these classical experiments that define the chondrocyte ‘phenotype', we performed RNA FISH on chondrocytes that were serially passaged in monolayer to induce ‘de-differentiation' and after subsequent ‘re-differentiated' in 3D culture (where one would expect a resumption of the cartilage phenotype)^35^,^36^.

For de-differentiation studies, we serially passaged chondrocytes nine times in monolayer with analysis at every other passage via RNA FISH. Consistent with classical findings^35^,^37^, the normalized ratio of aggrecan to GAPDH expression level decreased with passage number (Fig. 5b). However, and quite surprisingly, this change was not due to a decrease in absolute aggrecan copy number (Fig. 5c). Rather, aggrecan copy number showed a small but significant increase from passage 0 (initial plating) to passage 1, before returning to passage 0 mean copy number at later passages. In contrast, there was a rapid increase in mean GAPDH copy number over the first passage (increasing ∼fourfold) that remained at these elevated levels through additional passages (Fig. 5d). Previous studies from our group have shown that global transcription (including expression of GAPDH and many other abundant ‘house-keeping' genes) correlates with and can be dictated by cell size^38^. We also found that chondrocyte spread-cell area generally increased with passage number (Fig. 5f, Supplementary Fig. 4) and that the mean volume of suspended cells increased by ∼threefold between primary isolation (passage 0) and passage 5 (Fig. 5e). Taken together, these findings suggest that aggrecan expression does not decrease with chondrocyte de-differentiation and does not correlate with chondrocyte functional potential at the population level. Instead, normalization to housekeeping genes obscures relatively minor changes in aggrecan gene expression that occurs during chondrocyte ‘de-differentiation'. These single-cell data suggest that canonical markers of the chondrocyte phenotype do not accurately describe the molecular profile of de-differentiation.

To further explore how normalization may confound our interpretation of gene expression changes, we forced the re-differentiation of culture-expanded chondrocytes that had lost their ‘phenotype'. To do so, we encapsulated chondrocytes at early and late passage (passage 0 and 5, respectively) in 3D agarose hydrogels, and monitored matrix synthesis and gene expression over 2 weeks via Alcian blue staining and RNA FISH (Fig. 5a). Consistent with classical studies^35^,^36^, early passage chondrocytes produced matrix robustly on encapsulation, while late passage (de-differentiated) chondrocytes showed a significant attenuation in matrix deposition (Fig. 5g). RNA FISH showed that after 1 day of agarose culture, late passage chondrocytes expressed more aggrecan and more GAPDH than early passage chondrocytes (Fig. 5h,i). Over 14 days, mean aggrecan levels were maintained in early passage cells, but decreased in late passage cells. In keeping with our findings in monolayer, the aggrecan/GAPDH ratio was strongly influenced by changes in GAPDH (Fig. 5j). These data further support the finding that absolute changes in aggrecan expression levels are not responsible for the loss of phenotype observed in serially passaged chondrocytes.

## Discussion

In this work, quantitative single-cell analysis of gene expression provided evidence that the abundance of mRNA markers is only weakly linked to the chondrogenic phenotype of cartilage and progenitor cells. Specifically, we found that both MSCs and chondrocytes exhibited rampant transcriptional heterogeneity. This observation was not altogether surprising for MSCs, given that a single MSC population is comprised of a heterogeneous pool of related but distinct clonal populations. However, the transcriptional heterogeneity within individual MSC colonies suggested that this overall population heterogeneity is not entirely due to the mixing of clonal populations of varying potency, but instead likely arose from random transcriptional processes. While such heterogeneity may confound the interpretation of ensemble measurements, if this variation reflected intrinsic differences in differentiation capacity or differentiated state, then it might be harnessed towards a productive end. That is, cell sorting based on this variability could enable selection of ‘superior' sub-populations for therapeutic applications. For example, the expression of ‘stemness markers' such as SOX2 (ref. 39), OCT4 (ref. 40) and NANOG^41^ can distinguish pluripotent cells from larger heterogeneous populations, and the expression of an early osteogenic marker enables enrichment of the stromal vascular fraction for osteogenic cells^15^. However, our data show that for naive MSCs, neither genome-wide transcriptional metrics nor the transcriptional abundance of MSC stemness and chondrogenic markers correlate with the ultimate functional capacity. Strikingly, the most predictive genes (*aggrecan* and *MMP13*) were negatively associated with chondrogenic capacity, potentially suggesting that high-transcriptional promiscuity in naive MSCs reflects an inability to undergo robust lineage commitment. Furthermore, our single-cell studies showed that while naive MSCs and chondrocytes represent opposite ends of the differentiation spectrum, their absolute expression of canonical differentiation markers largely overlapped. When we monitored gene expression and cartilage-like matrix accumulation simultaneously on a cell-by-cell basis, marker expression taken at a single time point only weakly associated with cell output of extracellular matrix. Thus, we conclude that marker expression would only enable a slight enrichment of the population (∼35% increase in high-performing cells over the unsorted population) while drastically restricting available cell number for therapeutic application.

One possible explanation of the disconnect between an individual cell's transcript abundance and differentiated state is that, for many genes, transcription is a stochastic process comprised of long ‘silent' periods punctuated by short transcriptional bursts^42^,^43^,^44^,^45^,^46^,^47^. Bursting kinetics are strongly dependent on both the gene in question and the stimulus that is applied^42^,^46^,^48^,^49^, along with the position in cell cycle and cell volume^38^,^50^. For instance, stimulation (for example, TGFβ) can induce a synchronized initial burst of target gene expression, but subsequent bursts are typically asynchronous^51^,^52^,^53^,^54^. Thus, two cells with fluctuating but equivalent gene expression over time may exhibit different copy number when sampled at a single time point. As recently reported^53^,^55^, the rate of fluctuation (slow versus fast) of a single gene manifests in the heterogeneity observed between and within small clonal clusters. Our findings of high intra-colony variability and sister cell divergence in MSCs suggest that marker copy number fluctuates rapidly over a short timescale. As a result, absolute marker gene expression is not strongly heritable in MSCs, and we speculate that cells sorted on the basis of such expression will undergo transcriptional shifts over time and with further population expansion. In other systems, such stochastic variation in gene expression not only marks but can also determine cell fate^56^,^57^,^58^,^59^,^60^,^61^,^62^. Here it is surprising that for aggrecan, a gene whose product plays such a critical role in the extracellular matrix, such emergent heterogeneity in transcript abundance does not appear to reflect true variation in potency.

The disconnect between expression and functional capacity (matrix accumulation) may also reflect the time history of the system and the influence of other regulatory mechanisms. Aggrecan core protein undergoes co- and post-translational modifications, and may be subject to processing or secretory errors^26^,^63^. It may be that not every cell that produces core protein can appropriately modify the core and secrete it into the extracellular space. Furthermore, integration and retention of aggrecan core protein within the extracellular matrix relies on association with the hyaluronic acid and collagen network and other molecules^26^,^64^, and even aggrecan that has been integrated into the established matrix may ultimately be degraded by aggrecanases produced locally^65^. Deficiencies in any of these steps could decouple even temporally constant aggrecan mRNA expression from aggrecan core protein accumulation in the pericellular space. However, our transcriptome-wide data suggest that there is not a transcript level correlation between functional capacity and any of the genes involved in these processing steps.

Collectively, our findings in MSCs show that instantaneous aggrecan expression is only tenuously connected to matrix deposition. Moreover, differentiation of these cells fails to recapitulate the potential of native chondrocytes and does not prevent the expression of markers of alternate lineages even at the single-cell level. Our finding that chondrocyte expression of aggrecan does not decrease with de-differentiation also supports this weak connection, and raises questions as to the role of marker gene expression in defining phenotype. While aggrecan is one of the most conventional markers for the cartilage phenotype, its absolute expression did not correlate with cartilage-like matrix production and did not change as cells ‘de-differentiated'. If aggrecan expression does not change, other elements of the cell must be responsible for shifting cell fate and altering the transcriptional ‘focus' of the cell. Here our finding of major shifts in GAPDH with minor changes in aggrecan during de-differentiation suggest that de-differentiation may be better characterized as a shift in cell focus rather than a loss in specific programmatic expression of marker genes. While it is not yet clear what cell-wide changes drive this process, future work utilizing transcriptomics may identify a more comprehensive set of markers that are predictive of differentiated cell function. Until phenotype and its basis in gene expression are more precisely defined, our results suggest that it may be ineffective to design therapies that seek to bolster phenotype by increasing expression of individual genes or regulating transcriptional control of individual promoter regions, even for those genes whose products are directly related to functional matrix assembly. Simply increasing the raw RNA signal available to the cell may be insufficient, and it may also be necessary to alter the transcriptional context in which this occurs. These findings challenge the traditional notion that marker gene expression defines or is even strongly associated with the chondrocyte phenotype, and identify new directions in progenitor cell biology to establish, enforce and select subpopulations for therapeutic application.

## Methods

### Cell isolation and expansion

MSCs were isolated from the tibial and femoral bone marrow of juvenile bovine cows (3–6 months, Research 87, Boylston, MA) and expanded in a basal media consisting of high-glucose DMEM with 10% FBS and 1 × antibiotic–antimycotic. After the initial plating reached ∼80% confluence, cells were passaged at a ratio of 1:3 before use in experiments. For single-cell-derived colonies, bovine MSCs were isolated as described above and seeded sparsely onto glass coverslips. Individual colonies were allowed to expand for 3 days in basal media, followed by 4 days in chondrogenic induction media before fixation. All cells in each colony were manually located and imaged as described below. Chondrocytes were isolated from articular cartilage from the trochlear groove of juvenile bovine knees. Cartilage was digested in basal media supplemented with type II collagenase (0.5 mg ml^−1^, Sigma-Aldrich) for up to 18 h. Isolated cells were filtered, washed and plated in basal media. To improve cell yield for chondrocyte re-differentiation studies, cartilage was also digested in basal media with pronase (2.5 mg ml^−1^, Calbiochem) for 1 h before collagenase digestion. For all studies, chondrocytes were expanded in basal media and passaged 1:10 when plates reached ∼80% confluence. All bovine cells were derived from animals used in the food production industry, and so no institutional approvals were required.

### Cell encapsulation

For 3D culture, MSCs (passage 2) or chondrocytes (passage 0 and passage 5) were encapsulated in 2% agarose microgels at a density of two million cells per ml. Molten 4% w/v agarose (type VII, Sigma, 44 °C) was mixed 1:1 with cells suspended in media and pipetted into small drops in a well plate. Round coverslips were placed on top of the molten drops to spread the mixture before the gel solidified, resulting in the formation of uniform microgels that were 10–12 mm in diameter (depending on coverslip diameter) and ∼400 μm thick. Coverslips were removed from the microgels before culture. Microgels were supplied with fresh media every 3 days and 24 h before collection. MSC microgels were maintained in a chemically defined media consisting of high-glucose DMEM supplemented with 1 × antibiotic–antimycotic, 40 ng ml^−1^ dexamethasone, 50 μg ml^−1^ ascorbate 2-phosphate, 40 μgml^−1^ L-proline, 100 μg ml^−1^ sodium pyruvate, 1.25 mg ml^−1^ bovine serum albumin, 5.35 μg ml^−1^ linoleic acid and 1 × insulin–transferrin–selenous acid premix (Corning CB-40350), either with or without 10 ng ml^−1^ TGFβ3 (R&D Systems)^66^. Chondrocyte microgels were cultured in basal media (high-glucose DMEM+10% FBS+1 × antibiotic–antimycotic) supplemented with 50 μg ml^−1^ ascorbate 2-phosphate. At defined time points, gels were fixed for 30 min in paraformaldehyde (PFA) and stored in 70% ethanol at 4 °C.

Cell viability in gels was assessed using the LIVE/DEAD Cell Viability Assay Kit (Molecular Probes L-3224). A custom Matlab script quantified the number of live (calcein-AM positive) and dead (ethidium-homodiner-1 positive) cells in three 4 × fields of view per microgel. To assess viability in conjunction with RNA FISH, a fixable, amine-binding green fluorescent dead cell stain (Molecular Probes L-23101) was employed. For fixable dead staining, microgels were washed with PBS, stained for 30 min in a 1:5,000 dilution in PBS, washed with PBS again, and then fixed in PFA before RNA FISH analysis, as described below.

### Chondrogenic pellet culture and biochemical content

Clonally derived passage 2 MSCs were formed into cell-rich pellets via centrifugation (200,000 cells per pellet) and cultured in chondrogenic induction media with TGFβ for 21 days^67^. Pellets were papain digested and biochemically assayed for glycosaminoglycan and DNA content using via the1,9-dimethylmethylene blue and Picogreen (Molecular Probes, Eugene, OR) assays, respectively^67^.

### Live cell imaging and tracking

To investigate mRNA levels as a function of the time history of division, passage 2 MSCs were seeded into two-well LabTek chambered coverglass dishes (Fisher Scientific) and cultured in chondrogenic induction media with TGFβ for 4 days. Seeded cells were supplied with fresh media every 3 days and 24 h before fixation. Over the last 3 days of culture, live cells were imaged using a Nikon Ti-E microscope with a custom environmental chamber. Transmitted light images were automatically acquired every 30 min over a period of 70 h using a × 10 air objective over a 289-image grid in each well of the two-well coverglass. Cell division was tracked manually using ImageJ, and matched to the corresponding RNA FISH quantification that followed.

### RNA fluorescence *in situ* hybridization and imaging

Single-molecule RNA FISH was performed on samples^18^. Microgels and monolayer cells were fixed in PFA and permeabilized with 70% ethanol before *in situ* hybridization was performed using the specified pools of oligonucleotides. Monolayer and microgel samples were simultaneously co-stained with oligonucleotide probes for osteopontin labelled with Cy3, LPL labelled with Alexa 594, aggrecan labelled with Atto 647 N and GAPDH labelled with Atto700 (Stellaris oligonucleotides, Biosearch Technologies). See Supplementary Table 9 for a complete list of sequences of oligonucleotide probes used in this study. Subsequently, samples were washed with 2 × saline sodium citrate buffer (SSC) with 10% formamide (Ambion), and then 2 × SSC supplemented with DAPI (Molecular Probes D3571) to stain the cell nuclei. Monolayer cells cultured in coverglass chambers were submerged in 2 × SSC for imaging. Microgels were mounted in 2 × SSC and compressed between a coverglass and slide for imaging. Cells in the microgel and small colonies were imaged using a Leica DMI600B automated widefield fluorescence microscope equipped with a × 100 Plan Apo objective, a Pixis 1024BR cooled CCD (charge-coupled device) camera, a Prior Lumen 220 light source, and filter sets specific for each fluorophore. Images in each fluorescence channel were taken as a series of optical z-sections (0.5–0.7 microns per section) spanning the vertical extent of each cell. To prevent differences in viability between conditions from confounding interpretation of single-cell gene expression, the fixable dead cell stain was used to establish a GAPDH copy number of >10 mRNA as a threshold to identify live cells for inclusion in further analysis (Supplementary Fig. 1D). When this FISH analysis was applied to live-imaged cells, single plane scans were performed using a Nikon Ti-E microscope with a × 63 Plan Apo objective.

### Quantification of copy number from RNA FISH images

On collecting images of RNA FISH samples, cell boundaries were manually identified and RNA spots were counted and localized using custom software written in MATLAB^18^. For spot counting in FISH images from live cell tracking, each cell was tracked through the acquired time series, and sister cells manually matched, with care taken to note the time since last division.

### Quantification of extracellular matrix deposition

Extracellular aggrecan protein content was quantified by immunostaining. Briefly, after the final wash stages of the FISH protocol, samples were incubated with primary antibody (Abcam ab3778, 1:50 in PBS) at 4 °C overnight, washed for 30 min in PBS, incubated with Alexa 488 secondary antibody (Invitrogen, 1:200 in PBS) at room temperature for 1 h, washed with PBS for 30 min and then mounted for imaging. For immunofluorescence images, a scorer blinded to the RNA FISH images examined the DAPI, GFP (aggrecan core protein) and transmitted light images to classify cells with and without extracellular aggrecan core protein staining.

Receiver operating characteristic curves were constructed and analysed using the pROC package in R^68^. Matrix deposition (high versus low) was used as the binary outcome, and sensitivity and specificity were calculated for possible thresholds of RNA copy number, a linear combination of RNA counts and RNA ratios. To construct the linear combination, the data sets corresponding to each batch (Batch 1: Donors D-F, assayed for aggrecan, osteopontin, LPL, GAPDH; Batch 2: Donors X-Z, assayed for COMP, Sox9 and GAPDH) were randomly split in half to create training and test data sets to be used for model construction and evaluation, respectively. Logistic regression was performed using glm in R, and non-significant terms were dropped. For batch 1, the final model was established as (equation (1)):


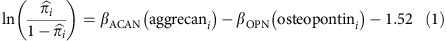


where 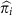, the estimated probability of the *i*-th cell having high matrix staining, was a function of the cell's aggrecan and osteopontin expression. Aggrecan associated positively (*β*_ACAN_=0.003, *P*<0.001), while osteopontin associated negatively (*β*_OPN_=−0.001, *P*=0.05); other markers were not significantly associated with matrix deposition. For batch 2, the intercept was the only significant term and the model was not further analysed. Having established this model on the training data set, its predictive performance was evaluated by constructing an ROC curve of the model applied to the test data set.

### RNA sequencing

Poly-adenylated RNA from passage 1 clonal MSCs populations were isolated from monolayer culture in basal media. The Qiagen miRNeasy kit was used for RNA isolation, the NEB Next Poly(A) mRNA Magnetic Isolation Module was used for selection of poly-adenylated transcripts and NEB Next Ultra Library Preparation Kit for Illumina was used for library preparation. Each sample was sequenced with 50-bp single-end reads on an Illumina HiSeq and to a depth of 15–25 M reads. Reads were aligned to bosTau7 using STAR^69^. Reads per gene were quantified using HTSeq and a RefSeq bosTau7 from annotation release 103 (ref. 70). FPKM (fragments per kilobase of transcript per million mapped reads) for each gene was calculated using R.

### Cell volume and area measurements

Chondrocyte area was measured in ImageJ by manually tracing images of phalloidin-stained cells sparsely plated onto glass coverslips. Chondrocyte suspended cell volumes were computed from the cell radii measured by an automatic cell counter (Nexcelom Cellometer) for chondrocytes in solution during passaging (immediately following trypsinization).

### Alcian blue staining

Microgels were removed from 70% ethanol and equilibrated in 3% acetic acid for 30 min at room temperature. Gels were then transferred to Alcian blue solution (pH 1.0, Rowley Biochemical) for 30 min, washed three times in acid alcohol (1% hydrochloric acid in 70% ethanol) for 30 min and then washed in PBS for 30 min before imaging. For macroscopic images, gels were photographed using a Ricoh photocopier and digital camera. For microscopic images, microgels were mounted in PBS and compressed between a coverglass and slide. Images were taken at × 10 using a Nikon Eclipse Ni-E motorized upright microscope.

### Statistical comparisons

To compare mean single-cell RNA counts, a generalized linear mixed model with a log-link function and by-donor random intercepts was constructed. For MSC RNA counts, media condition, and culture duration (with interaction term) were considered fixed effects, and an additional by-donor random slope effect was associated with media (−TGFβ versus +TGFβ). For MSC RNA divergence, time-since-division was considered a fixed effect. For chondrocyte RNA counts during de-differentiation in monolayer, passage was considered a fixed effect and was also associated with a by-donor random slope. For chondrocyte RNA counts during re-differentiation, passage and culture duration (with interaction term) were considered fixed effects, and an additional by-donor random slope effect was associated with passage. In each model, estimated means were compared using Satterthwaite-based t-distributions with simulated adjustment for multiple comparisons (SAS Studio 3.3). Pooled chondrocyte aggrecan/GAPDH expression data were compared using a one-way analysis of variance with Tukey's *post hoc* test. Chondrocyte area and volume data were pooled across donors and compared using a one-way analysis of variance with Tukey's *post hoc* tests. Sample size was chosen based on previous experience with these assays. Details of all statistical comparisons are provided in the Supplementary Tables.

## Additional information

**Accession codes:** RNA-Seq data have been deposited in NCBI GEO under accession code GSE76881.

**How to cite this article:** Cote, A. J. *et al*. Single-cell differences in matrix gene expression do not predict matrix deposition. *Nat. Commun.* 7:10865 doi: 10.1038/ncomms10865 (2016).

## Supplementary Material

Supplementary Information Supplementary Figure 1-4 and Supplementary Table 1-9.

## Figures and Tables

**Table 1:** Mean and coefficient of variation (CV) associated with aggrecan RNA count in undifferentiated and differentiated cells.

	**Mean aggrecan**	**Aggrecan CV**
Naive MSCs	69	1.60
Day 1 MSCs in gels	247	0.69
Day 21 MSCs in gels	334	0.72
Chondrocytes	225	0.57

MSC, mesenchymal stem cell.
