# Supplementary material for: Single-cell differences in matrix gene expression do not predict matrix deposition
Source: Nat Commun. 2016 Mar 3;7:10865. doi: 10.1038/ncomms10865 (PMC4782061; doi:10.1038/ncomms10865)
Supplement: Supplementary Information — Supplementary Figure 1-4 and Supplementary Table 1-9. [file ncomms10865-s1.pdf]

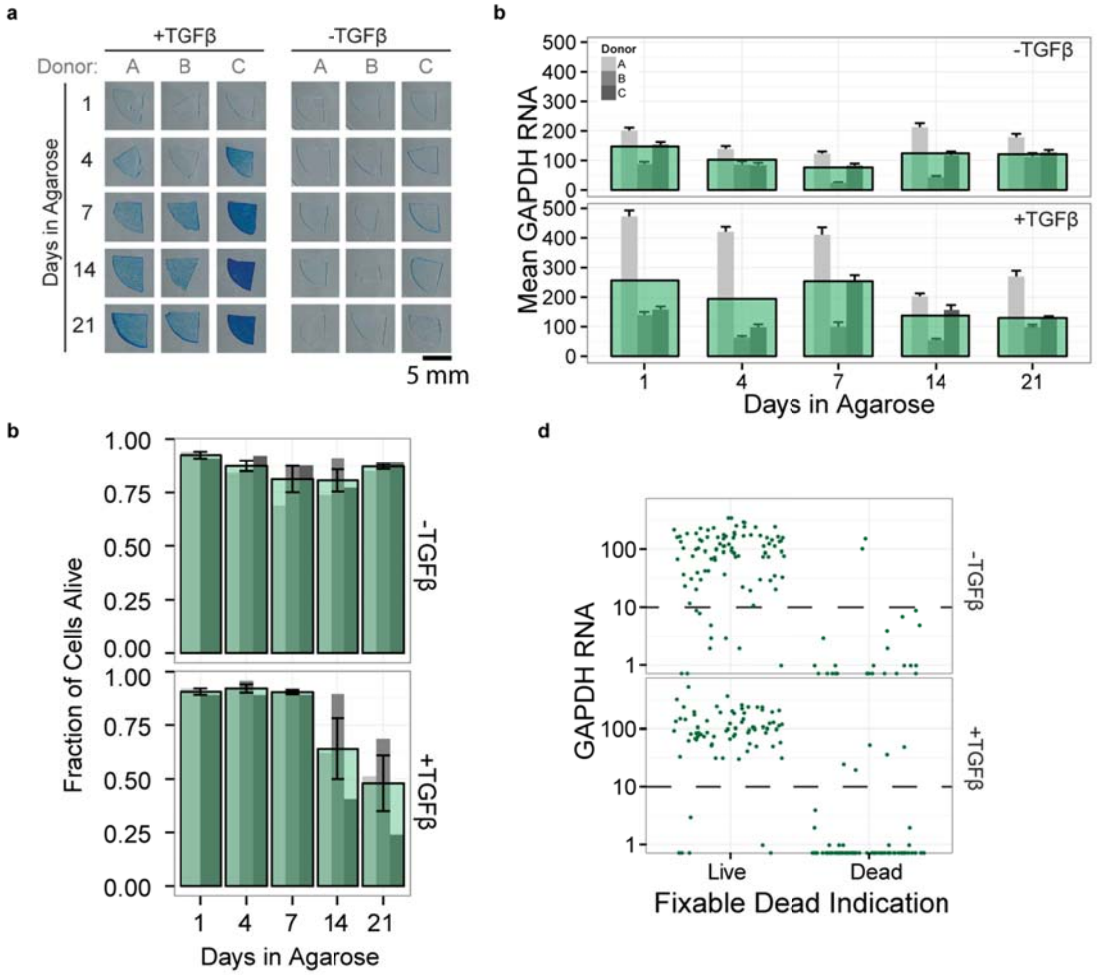

Supplementary Figure 1: Matrix production, GAPDH copy number, and viability of MSCs in 3D culture. a) Alcian blue staining for sulfated proteoglycans for MSCs in 3D culture, for all donors and time points, with and without TGFβ induction of chondrogenesis. Donor B is also shown in Figure 1. Scale bar = 5 mm. b) Mean GAPDH RNA counts and c) cell viability over 21 days in 3D culture. Narrow bars represent the mean within an individual donor; overlaid bars represent the mean across donors. Error bars indicate standard error (n = 24-128 cells per donor and condition). RNA count means compared by t-tests with Satterthwaite approximation and simulated adjustment for multiple comparisons. See Supplementary Table 1 for all statistical comparisons. d) Simultaneous RNA FISH and fixable dead staining established a threshold of GAPDH>10 to differentiate live cells from dead cells for further analysis. n = 85 cells for TGFβ-, 75 cells for TGFβ+.

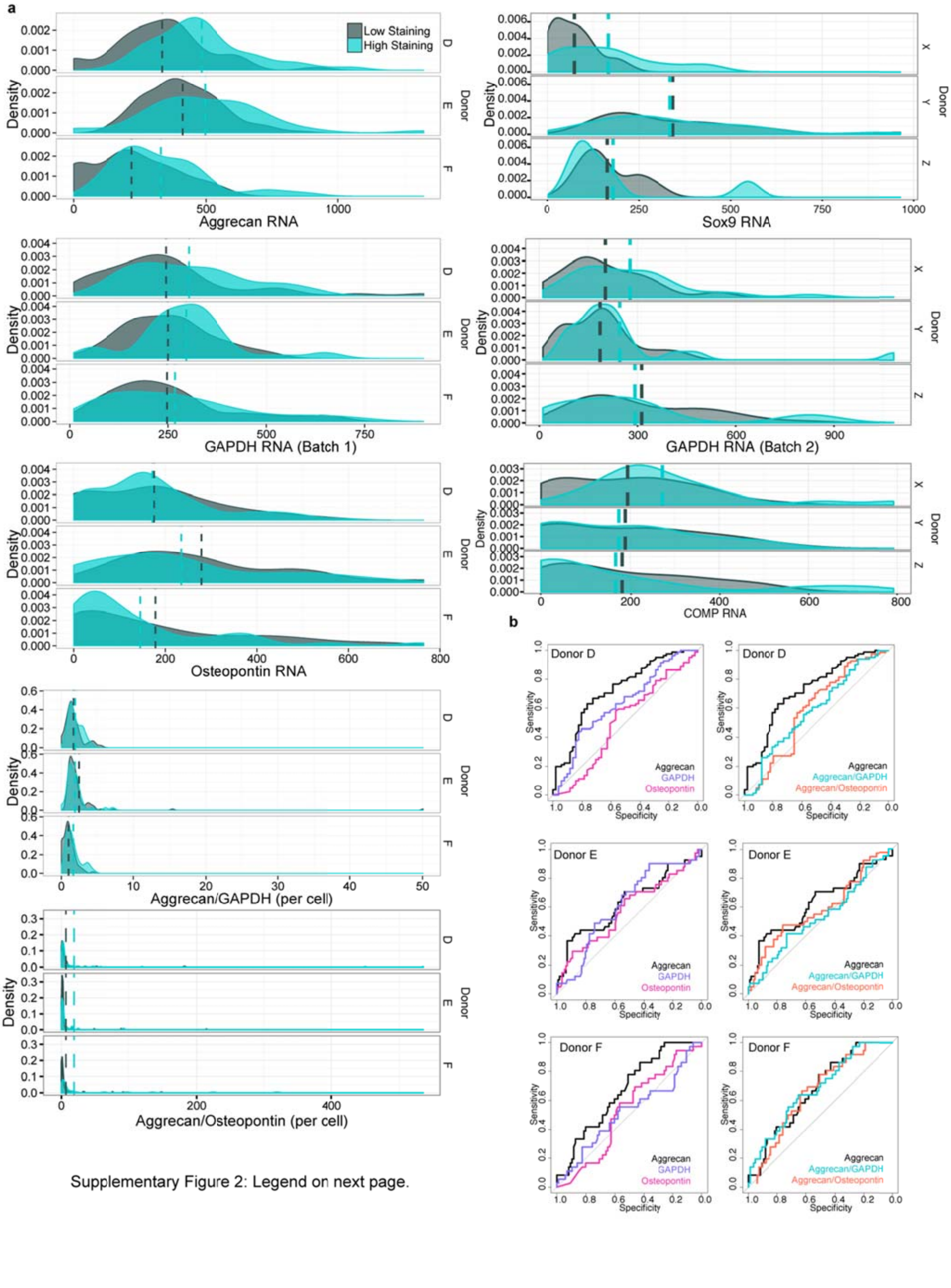

Supplementary Figure 2: Legend on next page.

Supplementary Figure 2: Matrix staining intensity versus mRNA copy number and ROC curves for individual donors, markers, and marker ratios.

a) Distribution of aggrecan, GAPDH, osteopontin, aggrecan/GAPDH, and aggrecan/osteopontin gene expression within high- and low-performing MSC populations; separated by donor. Dashed lines represent the mean for each condition. For aggrecan/osteopontin, only cells that had at least one osteopontin (>95% of cells per donor) mRNA were included in the graph and mean statistics calculation.

b) Receiver operating characteristic curves using individual gene expression and gene expression ratios to distinguish between high- and low-performing MSCs, separated by donor. Cells/donor: D: 132, E: 153, F: 122, X: 57, Y: 42, Z: 47.

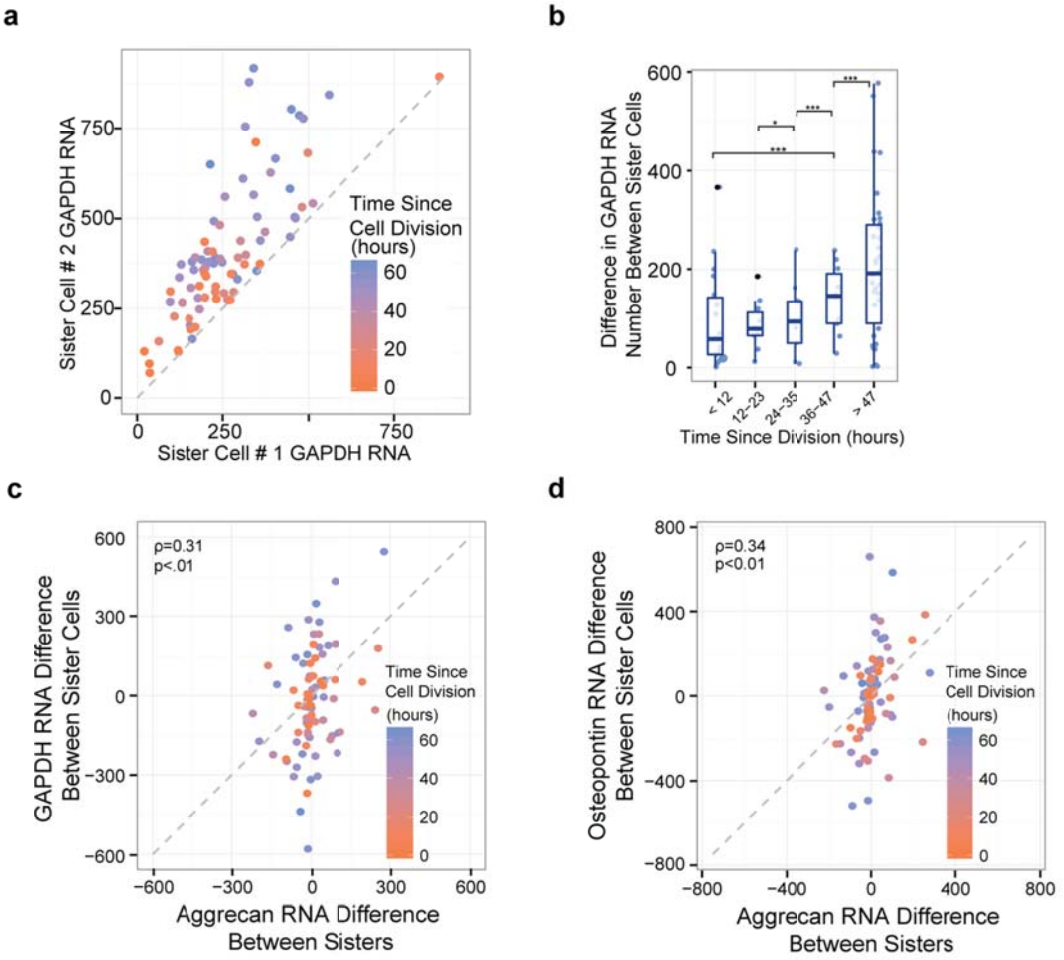

Supplementary Figure 3: Heritability of marker copy number through cell division.
a-b) Divergence in gene expression between sister cells as a function of time since their last division. c) Divergence in GAPDH vs aggrecan and d) osteopontin vs aggrecan between sister cells as a function of time since their last division. Box hinges denote the first and third quartiles. Whiskers extend from the hinges to the most extreme data points within (1.5 * interquartile range) of the hinges. Means compared by t-tests with Satterthwaite approximation and simulated adjustment for multiple comparisons, *** indicates $p<0.001$,* $p<0.05$. See Supplementary Table 6 for all statistical comparisons.
n = 81 sister cell pairs.

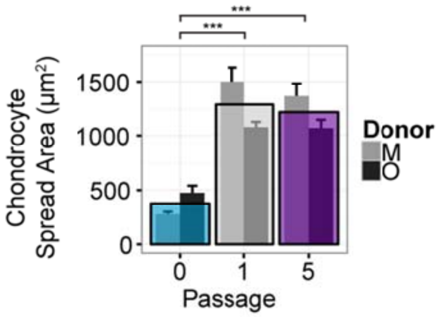

Supplementary Figure 4: Chondrocyte morphology with passage number.
Chondrocyte spread area with increasing passage number during de-differentiation (n = 25-27 cells per donor per condition). Narrow bars represent the mean within an individual donor; overlaid bars represent the mean across donors. Error bars indicate standard error. Compared via one-way ANOVA with Tukey post-hoc test, *** indicates $p<0.01$.

Supplementary Table 1	
**Comparison**	**Adjusted P-value**
**-TGFB vs +TGFB, Day 01**	0.3572
**-TGFB vs +TGFB, Day 04**	0.0827
**-TGFB vs +TGFB, Day 07**	0.0361
**-TGFB vs +TGFB, Day 14**	0.9479
**-TGFB vs +TGFB, Day 21**	0.9980
**+TGFB, Day 1 vs Day 4**	0.0022
**+TGFB, Day 1 vs Day 7**	0.4637
**+TGFB, Day 1 vs Day 14**	<.0001
**+TGFB, Day 1 vs Day 21**	0.0002
**+TGFB, Day 4 vs Day 7**	0.0019
**+TGFB, Day 4 vs Day 14**	0.0002
**+TGFB, Day 4 vs Day 21**	0.0009
**+TGFB, Day 7 vs Day 14**	<.0001
**+TGFB, Day 7 vs Day 21**	0.0002
**+TGFB, Day 14 vs Day 21**	0.0016

Supplementary Table 1: Additional statistical information related to GAPDH RNA abundance differentiating MSCs. Companion table to Supplementary Figure 1b. P-values indicated for all planned contrasts between –TGFβ and +TGFβ groups over 21 days of culture. GAPDH RNA count means compared by t-tests with Satterthwaite approximation and simulated adjustment for multiple comparisons.

Supplementary Table 2			
**Timepoint/Gene Pair**	**r^2**	**Pearson's correlation coefficient**	**p-value**
**Day 1 aggrecan - osteopontin**	0.24	0.49	1.0e-07
**Day 21 aggrecan - osteopontin**	.11	.34	.0019
**Day 1 aggrecan-LPL**	7.5e-5	.0087	.92
**Day 21 aggrecan-LPL**	.0022	.047	.67
**Day 1 osteopontin-LPL**	1.9e-5	.0044	.96
**Day 21 osteopontin-LPL**	.04	.21	.059

Supplementary Table 2: Correlation of marker gene RNA abundance in individual differentiating MSCs. r^2 and Pearson's correlation coefficient between abundance of aggrecan, osteopontin, and LPL in MSCs cultured with TGFβ in agarose for either 1 or 21 days. Test for the significance of the Pearson correlation coefficient was conducted with 103 degrees of freedom for Day 1 comparisons, and and 77 degrees of freedom for Day 21 comparisons. n= 105 cells at Day 1 and 79 cells at Day 21.

Supplementary Table 3				
	**Colony A**	**Colony B**	**Colony C**	**Colony D**
**Aggrecan CV**	1.71	1.34	1.07	0.91
**GAPDH CV**	0.32	0.35	0.31	0.20

Supplementary Table 3: Coefficient of variation (CV) of single cell RNA count in small MSC colonies. GAPDH RNA abundance was less variable in small colonies than aggrecan RNA abundance. (n = 75 cells in colony A, 7 cells in colony B, 6 cells in colony C, 8 cells in colony D).

Supplementary Table 4	
**Comparison**	**Adjusted P-value**
**-TGFB vs +TGFB, Day 01**	0.0067
**-TGFB vs +TGFB, Day 04**	0.0043
**-TGFB vs +TGFB, Day 07**	0.0020
**-TGFB vs +TGFB, Day 14**	0.0017
**-TGFB vs +TGFB, Day 21**	0.0031
**+TGFB, Day 1 vs Day 4**	0.0006
**+TGFB, Day 1 vs Day 7**	0.0002
**+TGFB, Day 1 vs Day 14**	0.0047
**+TGFB, Day 1 vs Day 21**	0.0002
**+TGFB, Day 4 vs Day 7**	0.0004
**+TGFB, Day 4 vs Day 14**	0.0021
**+TGFB, Day 4 vs Day 21**	0.0002
**+TGFB, Day 7 vs Day 14**	0.0002
**+TGFB, Day 7 vs Day 21**	<.0001
**+TGFB, Day 14 vs Day 21**	0.0002

Supplementary Table 4: Additional statistical information related to aggrecan RNA abundance differentiating MSCs. Companion table to Figure 1e. P-values indicated for all planned contrasts between –TGFβ and +TGFβ groups over 21 days of culture. Aggrecan RNA count means compared by t-tests with Satterthwaite approximation and simulated adjustment for multiple comparisons.

Supplementary Table 5		
**Comparison**		**Adjusted P-value**
**< 12 hrs**	**[12-23 hrs]**	<.0001
**< 12 hrs**	**[24-35 hrs]**	<.0001
**< 12 hrs**	**[36-47 hrs]**	<.0001
**< 12 hrs**	**> 47 hrs**	<.0001
**[12-23 hrs]**	**[24-35 hrs]**	0.8759
**[12-23 hrs]**	**[36-47 hrs]**	0.7564
**[12-23 hrs]**	**> 47 hrs**	0.9997
**[24-35 hrs]**	**[36-47 hrs]**	<.0001
**[24-35 hrs]**	**> 47 hrs**	<.0001
**[36-47 hrs]**	**> 47 hrs**	<.0001

Supplementary Table 5: Additional statistical information related to divergence in aggrecan RNA abundance between sister cells. Companion table to Figure 4f. Means compared by t-tests with Satterthwaite approximation and simulated adjustment for multiple comparisons, n = 81 sister cell pairs.

Supplementary Table 6		
**Comparison**		**Adjusted P-value**
**< 12 hrs**	**[12-23 hrs]**	0.1649
**< 12 hrs**	**[24-35 hrs]**	0.6435
**< 12 hrs**	**[36-47 hrs]**	<.0001
**< 12 hrs**	**> 47 hrs**	<.0001
**[12-23 hrs]**	**[24-35 hrs]**	0.0173
**[12-23 hrs]**	**[36-47 hrs]**	<.0001
**[12-23 hrs]**	**> 47 hrs**	<.0001
**[24-35 hrs]**	**[36-47 hrs]**	<.0001
**[24-35 hrs]**	**> 47 hrs**	<.0001
**[36-47 hrs]**	**> 47 hrs**	<.0001

Supplementary Table 6: Additional statistical information related to divergence in GAPDH RNA abundance between sister cells. Companion table to Supplementary Figure 3b. Means compared by t-tests with Satterthwaite approximation and simulated adjustment for multiple comparisons, n = 81 sister cell pairs.

Supplementary Table7				
Passage Comparison		Aggrecan/GAPDH (Pooled Population) Adjusted P-value	Aggrecan per Cell Adjusted P-value	GAPDH per Cell Adjusted P-value
0	1	0.0079	0.2108	0.0006
0	3	0.0004	0.9342	0.0006
0	5	0.0002	0.9999	0.0008
0	7	0.0002	1.0000	0.0008
0	9	0.0011	0.9984	0.0025
1	3	0.7036	0.6621	1.0000
1	5	0.5040	0.2891	0.9945
1	7	0.4593	0.1658	0.9929
1	9	0.9377	0.3623	0.7026
3	5	0.9993	0.9783	0.9981
3	7	0.9982	0.8850	0.9972
3	9	0.9944	0.9934	0.7784
5	7	1.0000	0.9988	1.0000
5	9	0.9510	0.9999	0.9343
7	9	0.9309	0.9941	0.9431

Supplementary Table 7: Additional statistical information related to RNA abundance in chondrocytes during passage-induced de-differentiation. Companion table to Figure 5b-d. P-values indicated for all planned contrasts between chondrocytes at different passages (passages 0-9). Pooled aggrecan/GAPDH levels compared via one-way ANOVA with Tukey's post-hoc test. Single cell RNA count means compared by t-tests with Satterthwaite approximation and simulated adjustment for multiple comparisons.

Supplementary Table 8			
Comparison	Aggrecan per Cell Adjusted P-value	GAPDH per Cell Adjusted P-value	Aggrecan/GAPDH per Cell Adjusted P-value
**Day 1, P0 vs P5**	0.0016	0.0406	0.9983
**P0, Day 1 vs 14**	0.0897	0.0006	0.0176
**Day 14, P0 vs P5**	0.0002	0.6714	0.0588
**P5, Day 1 vs 14**	<.0001	<.0001	0.1982

Supplementary Table 8: Additional statistical information related to RNA abundance in chondrocytes during re-differentiation in agarose. Companion table to Figure 5h-j. P-values indicated for all planned contrasts between chondrocytes at different passages (passages 0 and 5) and culture time points (days 1 and 14). Pooled aggrecan/GAPDH levels compared via one-way ANOVA with Tukey's post-hoc test. Single cell RNA count means compared by t-tests with Satterthwaite approximation and simulated adjustment for multiple comparisons.

Supplementary Table 9						
**Target:**	**Aggrecan**	**GAPDH**	**Osteopontin**	**Lipoprotein Lipase**	**COMP**	**Sox9**
# probes:	32 probes	20 probes	24 probes	32 probes	32 probes	7 probes
Probe 1	gtccttgtctccatagcaac	agaattaaaagcagccctgg	cccaagaggcagaagcaaat	ggagtcctgagagcaaattt	gtaatctccttgacctgctg	aaggggtccaggagattcat
Probe 2	acacgtcataggtttcgttg	tcattgatggcgacgatgtc	gtttaactggaagggcggag	caggaatgaggtggcaagtg	catcaccgtgtttttcagga	ctgctcgtcggtcatcttca
Probe 3	cttggaaggtgaacttctcc	agaccatgtagtgaaggtca	tacagcatctgggtatttgt	tgctgtggttgaagtgacag	gttggctgtctcggtacaag	cttggggaacgtgttctcct
Probe 4	tttctgggatgtccacaaag	ttgactgtgccgttgaactt	aagtctgcttctgagatggg	caactctcatacattcctgt	ctggtattgatgcagcgaac	gacggcctcgcggatgcaca
Probe 5	cccttacttcagggacaaac	ctttccattgatgacgagct	gacacagaattctgtggtgc	gacactggataatgctgctg	cgtacaaacctgcttgttgg	cgtagcccttgagcacctgg
Probe 6	actgatgtcctctactccag	gatctcgctcctggaagatg	gggtattttgtttgttgtca	ataaacttggccacatcctg	cggtctcacactcgttaatg	ggcatgggcaccagcgtcca
Probe 7	gcagagatttctggaccttc	tctccatggtagtgaagacc	ggctttcattggacttactt	agttaaattcatccgccatc	attgacgcatacagagttgg	ttgttcttgctcgagccgtt
Probe 8	actgatgtcctctactccag	agagatgatgaccctcttgg	gtcatctagatcgtctgttt	atcccaagagatgcacattg	gaaagccgtctaagtctgtg	
Probe 9	aaggtcctctactccagaag	ttgttatacttctcgtggtt	cgtcggagtcattagagttg	ttcttattggtcagacttcc	cagttgtcctttctgcactg	
Probe 10	agaaggaagtccactaaggt	gaggcattgctgacaatctt	aatggtcagggtcatcagtg	catactcgaagttaggtcca	gcagttgtccttctcattga	
Probe 11	agtctgctgagatcctctac	ccaaagtggtcatggatgac	tcagaatggtgagactcgtc	caggagaaaggcgacttgga	cttgtcgccatccgtatttc	
Probe 12	tctactccagaagcagagac	aagcagggatgatattctgg	gcgattgttggaatatcagt	ccctggtgaatgtgtgtaaa	cattctggtcgtcgttcttc	
Probe 13	actgatgtcctctactccag	ttgagctcagggatgacctt	ccgtagggataaacggagtg	ttctggattccgatacttcg	ctttctgatctgagttgggc	
Probe 14	caccagaaggaagaacactg	gtcagatccacaacagacac	tcttgacttcagtccgtaag	cgttagggtaaatgtccaca	ttgtcacaggcatctcctac	
Probe 15	actgatgtcctctactccag	catacttggcaggtttctcc	ttagatcggcggaacttctt	caatgttacatcctggttgg	gtctgcgttgctcttctgag	
Probe 16	actgatgtcctctactccag	gtcctcagtgtagcctagaa	tgtggcatctggactctgaa	ctctgcaatcacacggagag	gtcttggtcgctgtcacaa	
Probe 17	cctccagaaggaagtctact	tgagtgtcgctgttgaagtc	ctgactcgtcttcttaggtg	taggccttacttggattttc	cagttgtccttcgagtcctg	
Probe 18	agaaggaagtccactaaggt	aatgagcttgacaaagtggt	tggcgtgagttctttggaaa	ggcagagacctttctcaaag	ccatcattgtcgtcatcgtc	
Probe 19	gaaggtattctgccaaggtc	ttgctgtagccgaattcatt	gaatgcttgttcttatcctt	tgaccttgttgatctcgtag	ctagacggcagttgtcccta	
Probe 20	gtccactgaaatcaggaagg	tactccttggaggccatgtg	tttcctgactctcaatcaga	tacatcttgctgcttctttt	accttgtccgcatcaaagtc	
Probe 21	caaagagtccagaggtttcc		aattcttggctgagtttgga	gcatctgagaacgagtcttc	ggacacacatcgatcttgtc	
Probe 22	ccactaatgtcaggaaaccc		atgatctaggtctagcttgt	cctggttggtgtatgtatta	acccagttagggtctatctg	
Probe 23	gtcacgccagatatttctcc		tttcaggtgtttgtcttctt	cagtgccatacagagagatc	atctccataccctggttgag	
Probe 24	gatttctggttgtccagagg		attgacctcagaagaggcac	aaaagggatgttctcgctct	gtcgctgttcatcgtctgta	
Probe 25	caattgctccagaagacagt			ttgtggaaacttcaggcagg	tggaacgtgccttcgaaatc	
Probe 26	aagttccagaatgcctgaag			aatatccacctccgtgtaaa	ggtagccaaagatgaaacca	
Probe 27	ccaaagatcccaaatggtct			ggaccagctgaagtaggaat	catgaccacatagaagctgg	
Probe 28	accactggattcaaaaagct			caccttttttgagtctctc	cagtacgtctgctccatctg	
Probe 29	tgggactgatgacacttcta			gataagacattttctcccgg	taggatgtcttgtccttcca	
Probe 30	cagatcagcttcatggaagg			tatcacaggtgactttcctt	cctcgtagaatcgcactcta	
Probe 31	ggattcgatttctagacgcg			acttgtcatggcatttcaca	catggtcgtatccaggatca	
Probe 32	attgatctcgtatcggtcct			cagccagactttctattcag	atgatgttctcctgggagaa	

Supplementary Table 9: RNA-FISH probe sequences.
Oligonucleotide probe sequences used to assay aggrecan, GAPDH, osteopontin, LPL, COMP and Sox9 RNA abundance.
